# Novel missense mutations in the glycine receptor β subunit gene (*GLRB*) in startle disease

**DOI:** 10.1016/j.nbd.2012.12.001

**Published:** 2013-04

**Authors:** Victoria M. James, Anna Bode, Seo-Kyung Chung, Jennifer L. Gill, Maartje Nielsen, Frances M. Cowan, Mihailo Vujic, Rhys H. Thomas, Mark I. Rees, Kirsten Harvey, Angelo Keramidas, Maya Topf, Ieke Ginjaar, Joseph W. Lynch, Robert J. Harvey

**Affiliations:** a Department of Pharmacology, UCL School of Pharmacy, 29-39 Brunswick Square, London WC1N 1AX, United Kingdom; b Institute for Structural and Molecular Biology, Department of Biological Sciences, Birkbeck College, London WC1E 7HX, United Kingdom; c Queensland Brain Institute, The University of Queensland, Brisbane, QLD 4072 Australia; d Institute of Life Science, College of Medicine, Swansea University, Swansea SA2 8PP, United Kingdom; e Wales Epilepsy Research Network, College of Medicine, Swansea University, Swansea SA2 8PP, United Kingdom; f Center for Human 2333 ZC Leiden, and Clinical Genetics, Leiden University Medical Center, Einthovenweg 20, The Netherlands; g Department of Paediatrics, Imperial College London, Hammersmith Hospital Campus, Du Cane Road, London W12 0HS, United Kingdom; h Department of Clinical Genetics, Sahlgrenska University Hospital, SE-41345, Gothenburg, Sweden; i School of Biomedical Sciences, The University of Queensland, Brisbane, QLD 4072, Australia

**Keywords:** *GLRA1*, *GLRB*, Glycine receptor, Hyperekplexia, Startle disease

## Abstract

Startle disease is a rare, potentially fatal neuromotor disorder characterized by exaggerated startle reflexes and hypertonia in response to sudden unexpected auditory, visual or tactile stimuli. Mutations in the GlyR α_1_ subunit gene (*GLRA1*) are the major cause of this disorder, since remarkably few individuals with mutations in the GlyR β subunit gene (*GLRB*) have been found to date. Systematic DNA sequencing of *GLRB* in individuals with hyperekplexia revealed new missense mutations in *GLRB*, resulting in M177R, L285R and W310C substitutions. The recessive mutation M177R results in the insertion of a positively-charged residue into a hydrophobic pocket in the extracellular domain, resulting in an increased EC_50_ and decreased maximal responses of α_1_β GlyRs. The *de novo* mutation L285R results in the insertion of a positively-charged side chain into the pore-lining 9′ position. Mutations at this site are known to destabilize the channel closed state and produce spontaneously active channels. Consistent with this, we identified a leak conductance associated with spontaneous GlyR activity in cells expressing α_1_β^L285R^ GlyRs. Peak currents were also reduced for α_1_β^L285R^ GlyRs although glycine sensitivity was normal. W310C was predicted to interfere with hydrophobic side-chain stacking between M1, M2 and M3. We found that W310C had no effect on glycine sensitivity, but reduced maximal currents in α_1_β GlyRs in both homozygous (α_1_β^W310C^) and heterozygous (α_1_ββ^W310C^) stoichiometries. Since mild startle symptoms were reported in W310C carriers, this may represent an example of incomplete dominance in startle disease, providing a potential genetic explanation for the ‘minor’ form of hyperekplexia.

## Highlights

► We report novel missense mutations in the GlyR β subunit gene causing startle disease. ► Mutation M177R in the extracellular domain decreases GlyR agonist affinity. ► Mutation L285R in TM2 produces spontaneously active channels. ► Mutation W310C in TM3 affects hydrophobic stacking and shows incomplete dominance. ► Mutations in *GLRB* have unique pathogenic mechanisms and modes of inheritance.

## Introduction

Inhibitory glycine receptors (GlyRs) are ligand-gated chloride channels enriched in the spinal cord, brainstem and retina, consisting of heteropentameric combinations of ligand-binding GlyR α subunits (α_1_–α_4_) together with the GlyR β subunit (Lynch, 2009). Each GlyR subunit is composed of an N-terminal extracellular domain and four α-helical membrane-spanning domains (M1–M4). M3 and M4 are linked by a long intracellular domain containing binding sites for a variety of intracellular factors. Although GlyR α and β subunits both play an active role in agonist binding (Dutertre et al., 2012; Grudzinska et al., 2005), the GlyR β subunit was, until recently, widely assumed to play solely a structural role in heteromeric GlyRs. In part, this was due to the key role of the GlyR β subunit in mediating high-affinity interactions with the synaptic clustering molecule gephyrin (Meyer et al., 1995), which in turn controls the dynamic localization of GlyRs at synaptic sites (Feng et al., 1998). More recently, the GlyR β subunit was also reported to interact with the proteins Vacuolar Protein Sorting 35 (Vps35) and Neurobeachin (Nbea), indicating a new role in GlyR trafficking (del Pino et al., 2011). However, the atypical M2 domain of the GlyR β subunit also confers resistance to the effects of picrotoxinin (Pribilla et al., 1994) and the insecticide lindane (Islam and Lynch, 2012) as well as influencing the main-state single-channel conductances of heteromeric αβ GlyRs (Bormann et al., 1993).

Initial cross-linking studies of affinity-purified native GlyRs suggested that heteromeric GlyRs exist in a 3α:2β subunit combination (Langosch et al., 1988). However, three innovative studies have recently revealed that the GlyR β subunit represents the major component of heteromeric GlyRs, which are more likely to exist in a 2α:3β stoichiometry. Grudzinska et al., 2005 compared the effects of mutations affecting predicted glycine binding residues in GlyR α_1_ and β subunits. Co-expression of mutant GlyR α_1_ with wild-type β subunits resulted in a full rescue of the EC_50_ value for glycine, whilst co-expression of wild-type GlyR α_1_ with mutant β subunits resulted in a decrease in EC_50_. A subsequent study (Dutertre et al., 2012) revealed that the GlyR β subunit contributes more to agonist binding site formation than had been previously realized. Experiments with GlyR α_1_-β subunit concatemers also revealed that functional heteromeric GlyRs can be produced when these were co-expressed with GlyR β subunit monomers but not when expressed alone, or with GlyR α_1_ subunit monomers (Grudzinska et al., 2005). This result was consistent with either a 2α:3β or a 1α:4β stoichiometry. However, quantification of (^35^S)methionine incorporation into recombinant α_1_β versus α_1_ subunit GlyRs suggested a 2α:3β stoichiometry and the subunit order β-α-β-α-β. This stoichiometry and subunit arrangement has recently been confirmed by imaging single antibody-bound GlyR α_1_β heteromers using atomic force microscopy (Yang et al., 2012).

Defects in the adult GlyR isoform (α_1_β) also have an important role in disease. Mutations in *GLRA1*, encoding the GlyR α_1_ subunit, are the major genetic cause of startle disease/hyperekplexia in humans (Chung et al., 2010; Shiang et al., 1993, 1995) and cause similar disorders in mice (Buckwalter et al., 1994; Holland et al., 2006; Ryan et al., 1994; Traka et al., 2006) and Poll Hereford cattle (Pierce et al., 2001). In humans, hyperekplexia affects newborn children and is characterized by exaggerated startle reflexes and hypertonia in response to sudden, unexpected auditory, tactile or visual stimuli. By contrast, *GLRB* mutations are less frequent, but recessive mutations have been discovered in three families with hyperekplexia (Al-Owain et al., 2012; Lee et al., 2012; Rees et al., 2002), in the mouse mutant *spastic* (Kingsmore et al., 1994; Mülhardt et al., 1994) and the zebrafish *bandoneon* mutant (Hirata et al., 2005). However, one conundrum is why so few *GLRB* mutations are found in startle disease relative to *GLRA1*, or *SLC6A5*, encoding the presynaptic glycine transporter GlyT2, which are the primary and secondary genetic causes of startle disease respectively (Carta et al., 2012; Giménez et al., 2012; Rees et al., 2006). Here we report the characterization of three new missense mutations in *GLRB*, resulting in M177R, L285R and W310C substitutions in the extracellular domain, and the M2 and M3 membrane-spanning domains, respectively. We present detailed molecular modeling and functional analysis of these new GlyR β subunit mutations, revealing novel pathogenic mechanisms and modes of inheritance.

## Materials and methods

### Patients and cases

49 individuals with a clinical diagnosis of hyperekplexia (non-habituating startle response, positive nose tap test, history of neonatal/infantile hypertonicity) but lacking mutations in *GLRA1* and *SLC6A5* were ascertained by referral from pediatricians from international centers. DNA analysis of *GLRB* was performed at the Laboratory for Diagnostic Genome Analysis (LDGA) of the Center of Human and Clinical Genetics of the Leiden University Medical Center. The LDGA is NEN-EN-ISO 15189:2007 accredited by the Dutch Accreditation Council. Informed consent for participation in the study and publication of clinical and genetic data was obtained by the referring clinicians (MV at Sahlgrenska University Hospital and FMC at Imperial College London).

### Genetic analysis

DNA was extracted from whole blood taken from affected individuals and relatives using a Gentra Puregene DNA purification Kit (Gentra Systems, Minneapolis, USA). Primers recognizing the ten *GLRB* coding exons and flanking splice sites were designed using the Lightscanner Primer Design Software package, version 1 (Idaho Technology Inc, Salt Lake City, Utah). To avoid allelic dropout, all primers were placed in intronic regions that were devoid of single-nucleotide polymorphisms (SNPs) as revealed by National Center for Biotechnology Information databases. Primer designs are provided in Supplementary table 1. Amplified PCR fragments were sequenced on an ABI PRISM 3730 using the BigDye Terminator cycle sequencing ready reaction kit (Applied Biosystems, Foster City, CA, USA) and analyzed using SeqScap software for comparative sequence analysis (Applied Biosystems). Single nucleotide variants (SNVs) were assessed against those found in NCBI dbSNP Build 136 and release 20100804 from the 1000 genomes project.

### Homology modeling of the human GlyR

Fold recognition of human GlyR α_1_ and β subunits was performed with HHPred (Söding et al., 2005), identifying the structure of the *Caenorhabditis elegans* glutamate-gated chloride channel (GluCl; PDB: 3RHW) (Hibbs and Gouaux, 2011) as the best template (p-value: 0). A profile–profile alignment between the human GlyR α_1_ subunit and the GluCl α subunit was generated using MUSCLE (Edgar, 2004) and T-COFFEE (Notredame et al., 2000) resulting in 44.4% sequence identity. A separate alignment was generated for the human GlyR β subunit and GluCl resulting in 38.9% sequence identity. A short part of the sequence at the N-terminus of the β subunit (residues 1–30), and the extended loop region between M3 and M4 of both the α_1_ and β subunits (residues 312–396 and 337–453, respectively) could not be accurately modeled due to lack of sequence identity and were therefore removed. The two alignments were inspected manually and combined to include all five subunits together to re-create the entire pentameric structure in the stoichiometry 2α_1_:3β with the subunit arrangement β-α-β-α-β. Using MODELLER-9v10 (Eswar et al., 2003), 50 models were built based on the combined alignment, restraining disulfide bonds between the cysteine residues involved in the cys-loops within each subunit (C138–C152 and C198–C209 in the α_1_ subunit and C161–C175 and C221–C233 in the β subunit). Each model was assessed by MODELLER with the Discrete Optimized Protein Energy (DOPE) statistical potential score (Shen and Sali, 2006) and the optimal model was selected based on the lowest score (normalized DOPE Z score: − 0.222). A further assessment of model quality was performed using QMEAN (Benkert et al., 2011) The QMEAN Z score for the human GlyR α_1_β model was − 3.43, indicating a reasonable model (Benkert et al., 2011). Selected non-synonymous substitutions were modeled into the GlyR homology model with the *swapaa* command in Chimera (Pettersen et al., 2004) using the Dunbrack backbone-dependent rotamer library (Dunbrack, 2002) and taking into account the lowest clash score, highest number of H-bonds and highest rotamer probability.

### Site-directed mutagenesis and expression constructs

Full-length human GlyR α_1_ and β subunits were cloned into the vector pRK5 as previously described (Chung et al., 2010). Mutations were introduced into pRK5-hGlyRβ using the QuikChange site-directed mutagenesis kit (Agilent). All expression constructs were confirmed by Sanger sequencing of the entire coding region.

### Cell surface biotinylation assays

Wild-type or mutant GlyR α_1_β heteromers were transiently-expressed in human embryonic kidney (HEK293) cells transfected using magnetofection (CombiMag: OZ Biosciences, Lipofectamine 2000; Invitrogen). GlyR α_1_ and β subunit expression constructs were transfected at a DNA ratio of 1:10 to promote the formation of heteromeric α_1_β GlyRs. 32 h after transfection, cell surface-expression of GlyR β subunits was measured using a cell membrane-impermeable reagent Sulfo-NHS-LC-Biotin (Pierce Biotechnology) as previously described (Chung et al., 2010). Proteins in the whole-cell lysates or cell-surface proteins were analyzed by Western blotting with an antibody against the GlyR β subunit (1:90; AbCam ab123886). An anti β-actin antibody (1:5000; AbCam) was used as a control to confirm that intracellular proteins were not labeled with biotin. The intensity of the immunoreactivity signal was quantified using ImageJ (http://rsbweb.nih.gov/ij/).

### Fluorescence-based imaging

Cells were imaged using an automated fluorescence-based screening system using EYFP^I152L^ fluorescence quench as an indicator of anion influx. In this technique, iodide flowing into the cell binds to and quenches EYFP^I152L^ fluorescence, thus providing an indication of the relative activity levels of membrane anion channels (Kruger et al., 2005). Briefly, HEK AD293 cells were transfected with the plasmid DNAs for wild-type and mutant GlyRs and with the DNA for EYFP^I152L^ as described in the results and plated into a 384-well plate. Unless otherwise indicated, all GlyR plasmid DNAs were transfected in equimolar ratios for both the fluorescence and patch-clamp electrophysiological assays. Within the following 24–32 h, the culture medium in the wells was replaced with extracellular solution (140 mM NaCl, 5 mM KCl, 2 mM CaCl_2_, 1 mM MgCl_2_, 10 mM HEPES, and 10 mM glucose, pH 7.4 using NaOH). After 30 min, fluorescence images of each well were obtained twice, before and after the application of NaI solution (140 mM NaI, 5 mM KCl, 2 mM CaCl_2_, 1 mM MgCl_2_, 10 mM HEPES, and 10 mM glucose, pH 7.4 using NaOH) containing varying concentrations of glycine. Values were pooled from 3 to 4 experiments with three wells each containing > 200 cells. To determine the glycine dose–response curve, an empirical three or four parameter Hill equation was fitted by a non-linear least squares algorithm using SigmaPlot 11.0 software. Throughout this study, ‘% quench’ is defined as the (initial fluorescence − final fluorescence) × 100 / initial fluorescence. Thus, a treatment that completely abolished all fluorescence would yield a 100% quench.

### Electrophysiology

Glycine-gated currents were measured using whole-cell patch-clamp electrophysiology at a holding potential of − 40 mV. Spontaneous single-channel currents were recorded from outside-out excised patches, held at − 70 mV and in the absence of agonist. HEK AD293 cells were transiently transfected with GlyR subunit expression constructs and plated on coverslips. Cells were continually superfused with extracellular solution (composition as above) and visualized with an inverted fluorescence microscope. Transfected cells were identified by co-transfection with an EGFP expression construct. Patch pipettes were pulled to final tip resistances of 1–4 MΩ (whole-cell) and 6–12 MΩ (outside-out) when filled with a standard intracellular solution (145 mM CsCl, 2 mM CaCl_2_, 2 mM MgCl_2_, 10 mM HEPES, 10 mM EGTA, pH 7.4 with CsOH). Whole-cell currents, which were filtered at 1 kHz and digitized at 2 kHz, were recorded using an Axon MultiClamp 700B amplifier and pClamp10.2 software. Single-channel currents, which were filtered at 5 kHz and digitized at 20 kHz, were recorded using an Axon Axopatch 200B and pClamp10.2 software. Drugs diluted in extracellular solution were applied to cells or patches via gravity-forced perfusion using parallel small tubes. Solution exchange was complete within 200 ms.

## Results

### Mutation analysis of GLRB in hyperekplexia

**Fig. 1:**
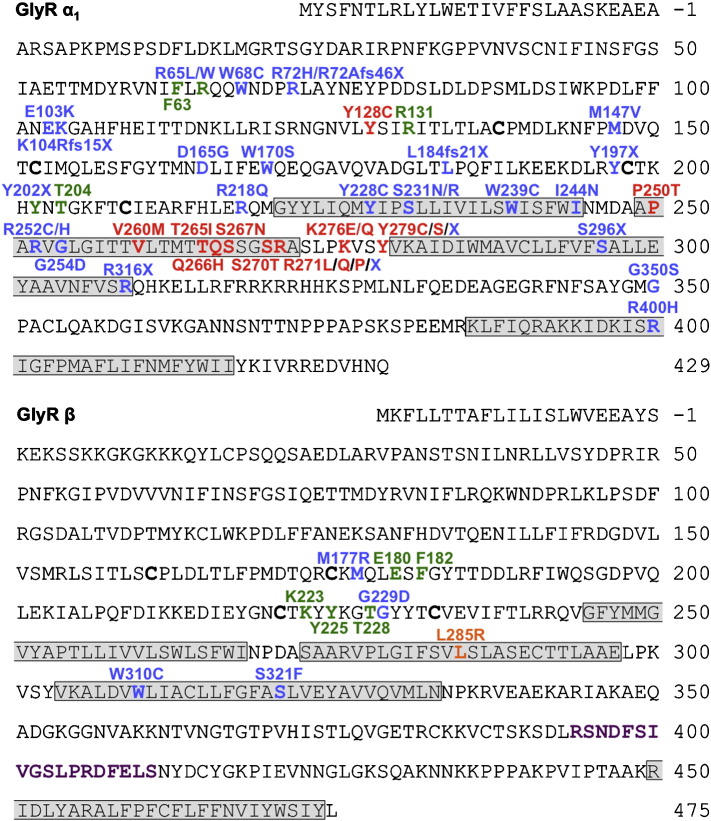
Human GlyR β subunit mutations identified in individuals with startle disease/hyperekplexia. Amino acid sequences of the human GlyR α_1_ and β subunits indicating the positions of putative membrane-spanning domains (grey shaded boxes) defined by alignments with GluCl (Hibbs and Gouaux, 2011) and amino-acid residues affected by hyperekplexia mutations. Mutation key: dominant mutation: red; recessive: blue; *de novo*: orange. Green lettering denotes key ligand-binding residues in the GlyR α_1_ and β subunits. Purple lettering denotes the gephyrin-binding motif between M3 and M4 of the GlyR β subunit.

Individuals with startle disease lacking mutations in *GLRA1* and *SLC6A5* were screened for genetic variation in *GLRB* coding exons and donor and acceptor splice junctions by Sanger DNA sequencing. Sequence variants were assigned as potentially pathogenic after cross-referencing with known *GLRB* mutations and common *GLRB* polymorphisms found in dbSNP and the 1000 genomes project. This analysis revealed two novel sequence variants in *GLRB* (Fig. 1A, B, Table 1) in two individuals. Individual 1 harbored a two base missense mutation (c.920_921ΔinsGA) resulting in a L285R substitution in the second membrane-spanning domain (M2) of the GlyR β subunit. Since this change was found in the heterozygous state (Fig. 1A, B) and neither parent carries the mutation, this mutation appears to be *de novo*. By contrast, individual 2 harbored a different missense mutation (c.G996T), resulting in a W310C substitution in the third membrane-spanning domain (M3) of the GlyR β subunit. At first glance, this mutation appeared to show recessive inheritance, since both parents were heterozygous carriers. However, clinical assessments and functional data suggest an alternative mechanism of inheritance for this mutation (see below). Recently, another recessive mutation c.T596G, resulting in a M177R substitution, was reported (Al-Owain et al., 2012) in nine patients in a large family of Saudi Arabian origin with hyperekplexia and esotropia, an eye misalignment disorder where one or both eyes turn inward.

### Clinical assessment

Individual 1 is a male of white British descent born at term plus 11 days. His weight and height were on the 3rd centile at delivery. Apneas were observed within 40 min and hypertonia presented within the first hour after birth. These symptoms were severe enough to necessitate intubation, his rigidity made ventilation very difficult and startle was prominent during this time. The classical nose-tap reflex was also present. Magnetic resonance imaging (MRI) revealed subtle brain abnormalities, including a mild increase in signal on T2-weighted sequences in the white matter and obvious dentate nuclei; however the basal ganglia had an unremarkable appearance. At 3 months of age stereotyped but unusual dystonic posturing movements were observed, including an arm-raising phenomenon that was followed by a period of generalized hypertonia during which voluntary movements were impossible. At 9 months, his gross motor performance was at the level of 7 months. Although this had broadly normalized by 14 months, dystonic movements were still prominent. Minor motor delay remained a feature for some time and when last seen he was still hypereflexic and had prominent cervical hypertonia, although exaggerated startle was less prominent; he also had an alternating strabismus. Now at 9 years, he remains small for his age. He still has very mild motor delay, but stiffness is not noticeable and he plays football regularly. He is making good progress in school and socially. He is mildly myopic but no longer has strabismus. No epileptic seizures have been observed.

Individual 2 is a female of Turkish origin with consanguineous parents. She exhibited neonatal hypertonia and irregular breathing necessitating continuous positive airway pressure (CPAP) treatment for several days following birth. In addition, she had episodes of neonatal bradycardia and an excessive startle reflex, although consciousness was unaltered during startle episodes. She had a short period of generalized stiffness following the startle response during which voluntary movements were impossible. MRI imaging performed at 2 months of age showed bilateral periventricular cystic changes, in keeping with periventricular leucomalacia (necrosis of white matter). She was not dysmorphic and is not currently of small stature, at only − 1 SD below the mean. At 4 months of age she had screaming periods without a clear loss of consciousness. Concurrently, she had spasms in the jaw and unusual movements of her arms. At that time her EEG was normal without focal signs. At 16 months of age she had an abnormal EEG with clear high-amplitude spike and wave activity and occasional hypsarrythmia (high amplitude and irregular waves and spikes in a background of chaotic and disorganized activity). She was treated with nitrazepam and both the seizures and EEG changes disappeared. She currently takes clonazepam and has no further symptoms. She does not have gaze palsy, eye movement disorders, nor any apparent learning difficulties. According to the family her parents and maternal uncle had ‘light symptoms’ in early life. However, further details of their early childhood have been lost over time and bound up within cultural restrictions surrounding disclosure. Her older sister (untested) has clear symptoms and her younger brother born in 2010 (tested) is also affected. The parents (both heterozygous for W310C) do not currently show any hyperekplexia symptoms.

### Molecular modeling

**Fig. 2:**
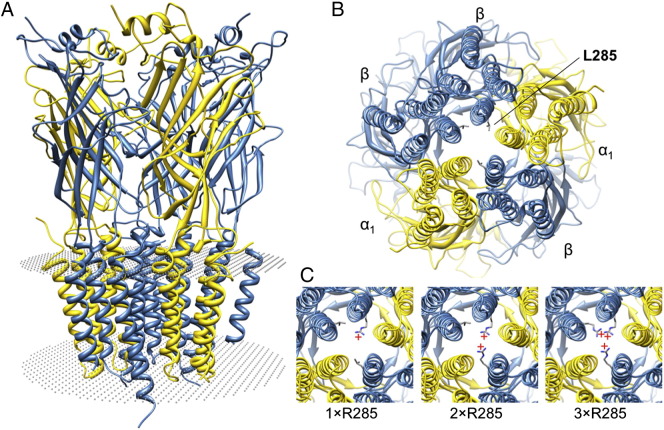
Molecular modeling of the human α_1_β GlyR and mutation L285R. (A) Side view of the molecular model of the human GlyR α_1_β pentamer showing α_1_ subunits in gold and β subunits in blue. The positions of the inner and outer membrane surfaces are indicated by grey spheres. (B) Orthographic view of the GlyR α_1_β pentamer, showing the relative position of the L285 side chain in the ion-channel pore. (C) Panels depicting the multiple GlyR isoforms that could be formed on co-expression of wild-type and mutant GlyR β subunits harboring the L285R substitution. A red plus sign denotes the positive charge carried by the side-chain of R285.

**Fig. 3:**
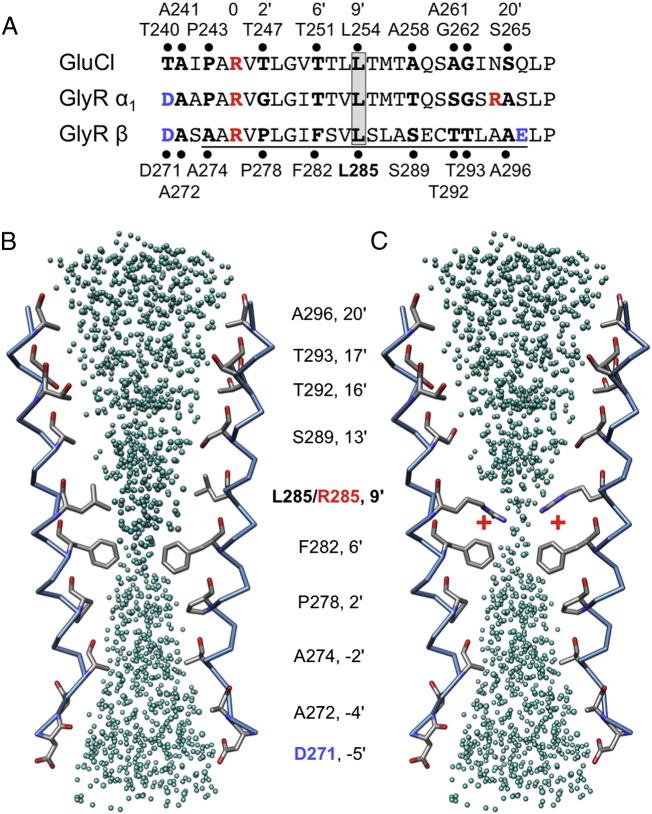
Mutation L285R inserts a charged residue into the GlyR ion-channel pore. (A) Sequence alignment of M2 of GluCl with GlyR α_1_ and β subunits. Relative positions of residues that line the Cl^−^ channel are indicated with black circles and residue numbers indicated for GluCl and the GlyR β subunit. Residues carrying presumed positive and negative charges are colored red and blue, respectively, although it should be noted that the protonation state of these residues at physiological pH has not been established. The position of L285 is indicated by grey highlighting. (B, C) Schematic diagrams depicting two GlyR β subunit M2 α-helices and residues lining the Cl^−^ channel for wild-type L285 (B) and the R285 mutant (C). Blue spheres represent the predicted internal volume of the ion channel and side-chains are shown for pore-lining residues. The L285R substitution is predicted to create a premature narrowing of the pore at the 9′ position and inserts a positively charged residue (+) into an α helix that is uncharged, with the exception of D271 on the cytoplasmic face of the ion-channel pore.

M177R, L285R and W310C substitutions were predicted (Table 1) to be damaging or probably/possibly damaging using SIFT (Kumar et al., 2009) and Polyphen-2 (Adzhubei et al., 2010), respectively. These bioinformatics tools predict the possible impact of an amino acid substitution on the structure using the conservation of amino acid residues in sequence alignments derived from related sequences and machine learning approaches. To gain further insights into the potential pathogenic mechanisms of these mutations, we constructed a homology model of the human α_1_β GlyR based on the crystal structure of the GluCl α subunit from *C. elegans* (Hibbs and Gouaux, 2011). The GluCl structure makes a significantly better template for homology modeling of the human GlyR compared to other ligand-gated ion channel structures such as *Gloeobacter violaceus* GLIC (Bocquet et al., 2009), *Erwinia chrysanthemi* ELIC (Hilf and Dutzler, 2009) or the *Torpedo marmorata* nAChR (Unwin, 2005) due to functional similarity (i.e. GluCl is an anion channel) and higher overall sequence identity. Profile–profile alignment between the human GlyR subunits and the GluCl α subunit generated using MUSCLE (Edgar, 2004) and T-COFFEE (Notredame et al., 2000) resulted in 44.4% (α_1_) and 38.9% (β) identity. By contrast, GlyR subunits share lower sequence identity with GLIC (α_1_: 21.8% and β: 24.6%) and different nAChR subunits (α_1_: 19.7 to 20% and β: 23.9 to 24.6%). Our GlyR model (Fig. 2A) predicts that L285 in the GlyR β subunit is a pore-lining residue, projecting a small uncharged, hydrophobic side chain into the ion-channel lumen (Fig. 2B). The L285R mutation introduces a significantly larger, positively-charged side chain that projects into the pore lumen (Fig. 2C). Because this mutation is *de novo* and the individual is heterozygous for L285R, in theory heteromeric GlyRs could be formed *in vivo* containing 3 × wild-type, 1×, 2 × or 3 × mutant GlyR β subunits (Fig. 2C). Modeling of pore-lining residues at a β subunit/β subunit interface (Fig. 3A, B) reveals that L285 is at the 9′ position in M2, equivalent to L254 in GluCl. This mutation is predicted to disrupt the pentameric radial symmetry of the 9′ leucine ‘hydrophobic girdle’, that is essential for stabilizing the pore in the closed channel state. Disruption of this girdle is well known to induce spontaneous channel activity and/or enhance agonist sensitivity in other Cys-loop receptors (Chang and Weiss, 1998, 1999). In common with GluCl, none of the major pore-lining residues are charged, with the exception of D271 at the -5′ position at the cytoplasmic end of M2 (Fig. 3A). This is consistent with different models of anion channel function which suggest that the positive electrostatic potential at the base of the pore arises from either: i) buried basic residues at the intracellular entrance of the pore (Cymes and Grosman, 2011), such as R276 in the M2 0′ position in the GlyR β subunit (R252 in the GlyR α_1_ subunit) or ii) oriented peptide dipoles in the M2 α helices (Hibbs and Gouaux, 2011). Given that the electrostatic potential at the 0′ position strongly influences single-channel conductance (Chang and Weiss, 1998, 1999), it is possible that the introduction of a large, positively-charged residue at the 9′ position could have a similar effect.

**Fig. 4:**
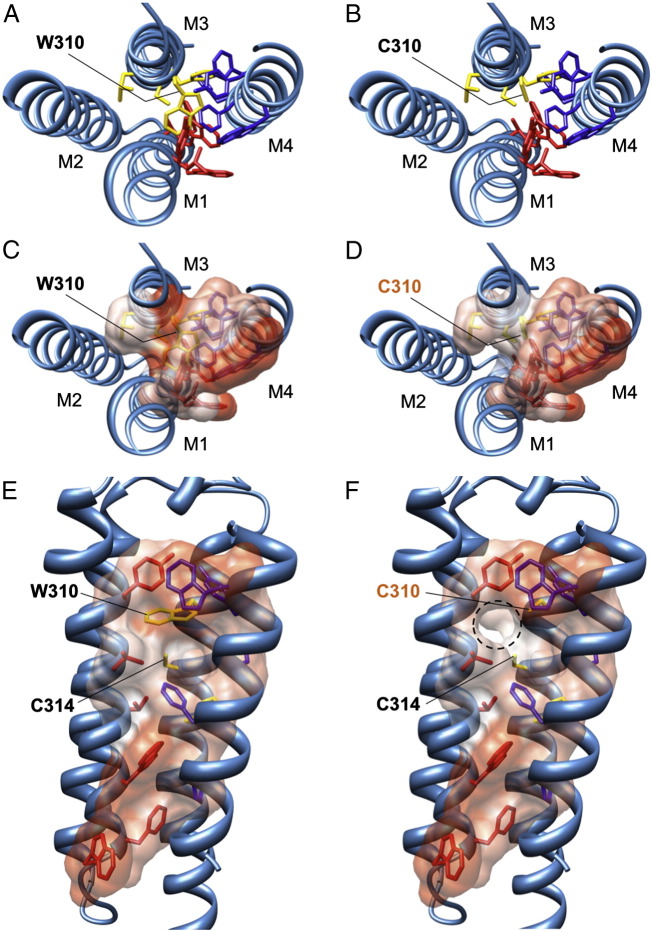
Mutation W310C disrupts hydrophobic stacking of membrane-spanning domains M1–M3. (A) Cutaway view from the extracellular side of the GlyR β subunit showing how the side-chain of residue W310 in M3 contributes to a hydrophobic stack of aromatic residues contributed by membrane-spanning domains M1, M3 and M4. Hydrophobic side-chains of residues predicted to be involved in this stack are colored according to membrane-spanning domain: M1: red; M3: yellow and M4: blue. (B) Mutation W310C results in the loss of the aromatic, hydrophobic side chain of tryptophan and replacement with the shorter, reactive cysteine sulfydryl side chain. (C–F) Top and side views showing the hydrophobicity of individual residues in the stack by coloring the atom surfaces. Hydrophobic surfaces are depicted in orange, hydrophilic in blue and white indicates neutral polarity. The W310C substitution is predicted to disrupt the hydrophobic stack, altering the tertiary fold of the membrane-spanning domains and introduce an empty space (F, circled with a dashed line). Disulfide bond formation is unlikely, since this is rare within the membrane or an intracellular environment and the only adjacent sulfur atom (C314 in M3) is 6.4 Å away from C310 sulfur.

By contrast, the side-chain of W310 in M3 is part of an intramembrane network of aromatic residues (phenylalanine, tyrosine, histidine and tryptophan) contributed by M1, M3 and M4 (Fig. 4A, B). Substitution W310C introduces a significantly smaller and shorter side chain into this position (Fig. 4C), with a reactive sulfhydryl group. Although we considered that C310 might form a disulfide bond with C314 in M3, one turn down the helix in our model, the distance between the two sulfur atoms (4.6 Å) precludes this interaction (Careaga and Falke, 1992). In addition, disulfide bonds rarely form within the membrane or a reducing intracellular environment. Rather, our modeling predicts that the W310C substitution disrupts aromatic stacking (Fig. 4B) — a key requirement for correct GlyR assembly and cell-surface trafficking (Haeger et al., 2010).

**Fig. 5:**
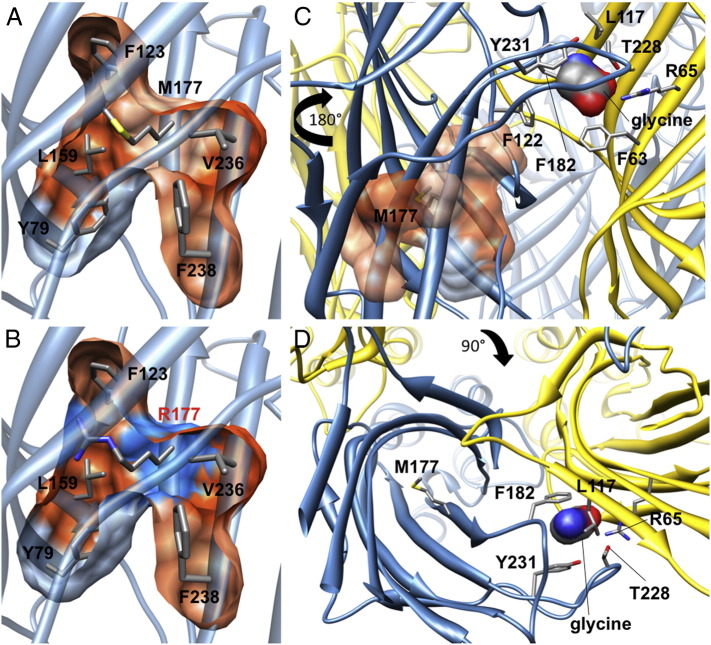
Mutation M177R disrupts local structure of a β-sheet in the N-terminal extracellular domain. (A, B) Side views of part of the GlyR β subunit extracellular domain showing the location of M177 (A) and R177 (B, red lettering) within a β-sheet structure. The polarity of the surface residues is shown with hydrophobic residues depicted in orange, hydrophilic in blue and white indicating neutral polarity. (C) Rotating the model 180° horizontally shows that M177 is located close to F182, which is involved in a cation-π interaction with the amino group of glycine. (D) Top view of M177, relative to the ligand-binding domain, rotated 90° vertically from B. Thus, mutation M177R is predicted to result in the loss of a hydrophobic side-chain and replacement with a positively-charged side chain. This substitution is predicted to disturb the local fold of the extracellular domain and have a knock-on effect for positioning of the critical ligand-binding residue F182.

M177 is a hydrophobic amino acid located in the GlyR extracellular domain on a β-sheet (β7). The side-chain of this residue forms part of a hydrophobic pocket (Fig. 5*A*) that is conserved within the GluCl structure (Hibbs and Gouaux, 2011), which has an isoleucine at the equivalent position. The M177R substitution results in the introduction of a positively-charged, hydrophilic side chain into this pocket (Fig. 5B), which is predicted to have a significant effect on the folding of the extracellular domain. Although M177/R177 are not predicted to directly influence glycine binding (Fig. 5C, D), disruption of the local structure around M177 is predicted to cause an indirect effect on agonist binding or signal transduction. For example, M177 is located just five residues away from F182, which is involved in a cation-π interaction (Pless et al., 2008) with the amino group of glycine.

### Cell-surface biotinylation assays

**Fig. 6:**
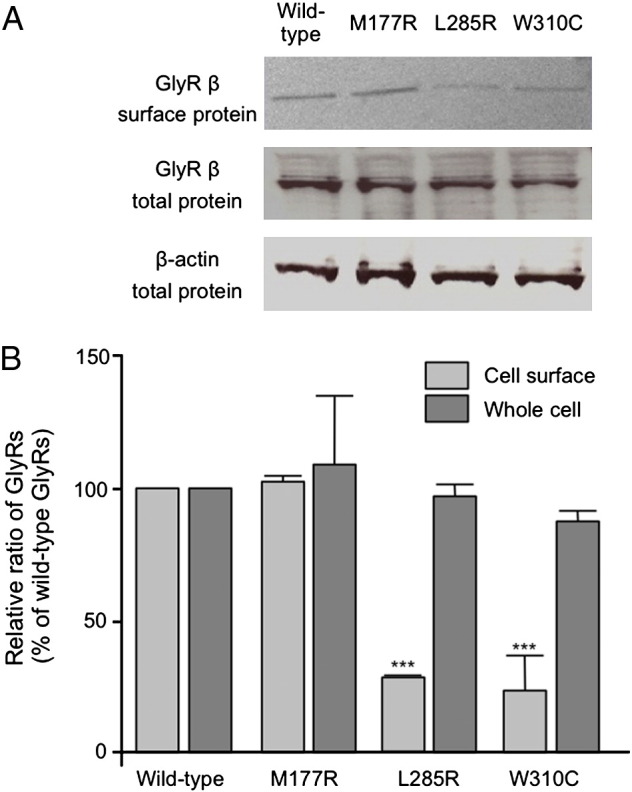
Cell-surface biotinylation assays for GlyR β subunit mutations. (A) Cell-surface biotinylation assays on heteromeric α_1_β GlyRs expressed in HEK293 cells revealed that all three GlyR β subunit mutants (M177R, L285R and W310C) were expressed at similar levels in whole-cell protein expression (total) compared to an actin control. However, mutations L285R and W310C revealed markedly reduced cell-surface protein expression (surface) compared to wild-type or the M177R mutant. 70 μg of protein lysates were loaded in the each lane. (B) Quantification of cell surface and whole cell expression of the GlyR β subunit mutants using ImageJ software, with values expressed as a percentage of wild-type GlyR β subunit expression. p-Values were calculated relative to wild-type GlyR β expression using an unpaired *t*-test; *** = p < 0.001.

To establish whether the GlyR β subunit mutants impaired cell-surface expression of GlyRs, we carried out cell-surface biotinylation assays for heteromeric α_1_β GlyRs (Chung et al., 2010), detecting β-subunit expression using a specific antibody. Note that in the absence of the GlyR α_1_ subunit, the GlyR β subunit is not expressed at the cell surface in HEK293 cells, but is retained in the rough endoplasmic reticulum (Kirsch et al., 1995). The M177R, L285R and W310C mutant polypeptides were all detected at equivalent levels to the wild-type GlyR β subunit in total cell lysates, suggesting that they synthesized correctly and are not prone to rapid degradation (Fig. 6*A*). However, quantification of the biotinylation of heteromeric cell-surface α_1_β GlyRs revealed significant decreases in cell-surface expression of α_1_β^L285R^ and α_1_β^W310C^ subunit GlyRs (Fig. 6A, B) to 27.3 ± 1.1% for L285R and 21.5 ± 8.3% for W310C of the level of wild-type α_1_β GlyRs. By contrast, α_1_β^M177R^ GlyRs had a similar level of cell-surface expression (104 ± 2.2%) when compared to wild-type α_1_β GlyRs.

### Functional analysis using EYFP Cl^−^ sensors and electrophysiology

**Fig. 7:**
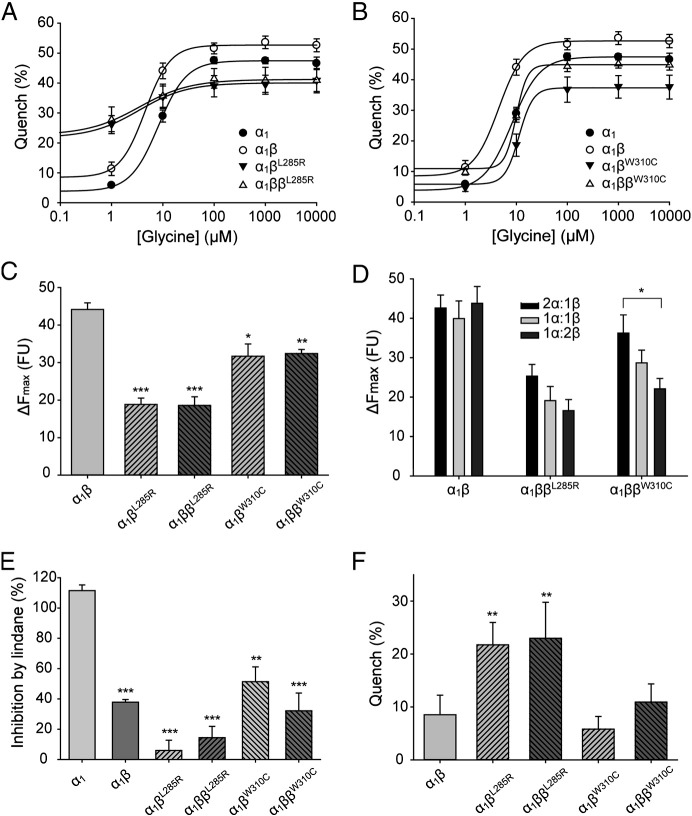
Functional characterization of human GlyR β^L285R^ and β^W310C^ mutations using the EYFP assay. (A, B) Mean glycine dose–response curves for α_1_, α_1_β, α_1_β^L285R^, α_1_ββ^L285R^, α_1_β^W310C^ and α_1_ββ^W310C^ GlyRs. The percentage quench is plotted against the applied glycine concentration (μM). Averaged parameters of best fit to dose–response curves are summarized in Table 2. (C) Maximal changes in fluorescence. ΔF_max_ is the initial fluorescence value minus the final fluorescence value and is represented in fluorescence units. p-Values were calculated relative to α_1_β GlyR heteromers by unpaired *t*-test: * p < 0.05, ** p < 0.01, *** p < 0.001. (D) Maximal changes in fluorescence for GlyR subunits transfected in different α:β ratios while the amount of α_1_ transfected was held constant. * p < 0.05 by unpaired *t*-test. (E) Inhibition by 100 μM lindane. The inhibition of currents activated by 10 μM glycine by 100 μM lindane is represented as a percentage reduction of the control maximal fluorescent quench. p-Values were calculated relative to GlyR α_1_ homomers by unpaired *t*-test: * p < 0.05, ** p < 0.01, *** p < 0.001. (F) Fluorescent quench in the presence of sodium iodide (no glycine). p-Values were calculated relative to GlyR α_1_β heteromers by unpaired *t*-test: * p < 0.05, ** p < 0.01, *** p < 0.001. All data points displayed in this figure represent the average quench from four experiments comprising three wells each with > 200 cells per well.

HEK AD293 cells were co-transfected with expression constructs encoding the GlyR α_1_ subunit together with each GlyR β subunit mutant. To reproduce the heterozygous state, GlyR α_1_ was co-transfected with wild-type and mutant GlyR β subunits. We initially employed the EYFP assay (Chung et al., 2010; Kruger et al., 2005) to provide an indication of the anion influx rate through the recombinant GlyRs. The advantage of this approach is that responses of large numbers of cells can be averaged, thus permitting the reliable quantitation of small changes in the current fluxing capacity of functional GlyRs. Glycine dose–response relationships (Fig. 7A, B) revealed that the maximal fluorescence quench was significantly reduced for α_1_β^L285R^, α_1_ββ^L285R^, α_1_β^W310C^ and α_1_ββ^W310C^ GlyRs relative to wild-type α_1_β GlyRs (Fig. 7C), suggesting that mutations L285R and W310C reduce anion flux rates in both the homozygous and heterozygous states. We also found that the reduction of Cl^-^ flux was dependent on the ratio of wild-type GlyR α_1_ subunit (the amount was held constant) to mutant GlyR β subunits (Fig. 7D), with significantly reduced Cl^-^ flux observed with increasing levels of β^W310C^ expression. Given that α_1_β GlyRs exist in an invariant 2α:3β stoichiometry (Yang et al., 2012), we infer that increasing the β^W310C^:α1 subunit ratio increases the proportion of heteromeric GlyRs, which in turn reduces functional GlyR expression. These data are consistent with our biotinylation assays, and suggest β^W310C^ subunits prevent wild-type GlyR subunits from trafficking to the cell surface. EC_50_ values for wild-type α_1_β, α_1_β^L285R^ and α_1_β^W310C^ GlyRs were 5 ± 1, 4 ± 1, 13 ± 2 μM, respectively (Table 2), suggesting that the L285R and W310C mutations do not substantially alter the agonist sensitivity of recombinant α_1_β GlyRs reaching the cell surface.

To test if the mutated β subunits incorporate into functional GlyRs, the pore blocker lindane was applied. Lindane potently inhibits homomeric α subunit GlyRs, but has almost no effect on heteromeric αβ subunit GlyRs (Islam and Lynch, 2012). GlyRs containing β^L285R^ or β^W310C^ subunits exhibited a reduced sensitivity to lindane, indicating that receptors incorporating these mutant subunits are functionally expressed at the cell surface (Fig. 7E). Since artificial mutations at the 9′ position in GABA_C_ and GABA_A_R subunits result in spontaneously-opening channels (Chang and Weiss, 1998, 1999), we also investigated whether α_1_β^L285R^ GlyRs showed this property, by quantifying the fluorescence change in the absence of glycine. Both α_1_β^L285R^ and α_1_ββ^L285R^ GlyRs showed a significant increase in quench relative to heteromeric α_1_β GlyRs, which is suggestive of spontaneous activity (Fig. 7F). Even in the absence of glycine, the quench observed was > 20%. By contrast, the quench observed in the absence of glycine for α_1_β^W310C^ containing GlyRs was only slightly higher than for wild-type GlyRs, suggesting that these GlyRs do not show spontaneous gating. Averaged results obtained using the EYFP Cl^−^ flux assay for L285R and W310C are summarized in Table 2.

**Fig. 8:**
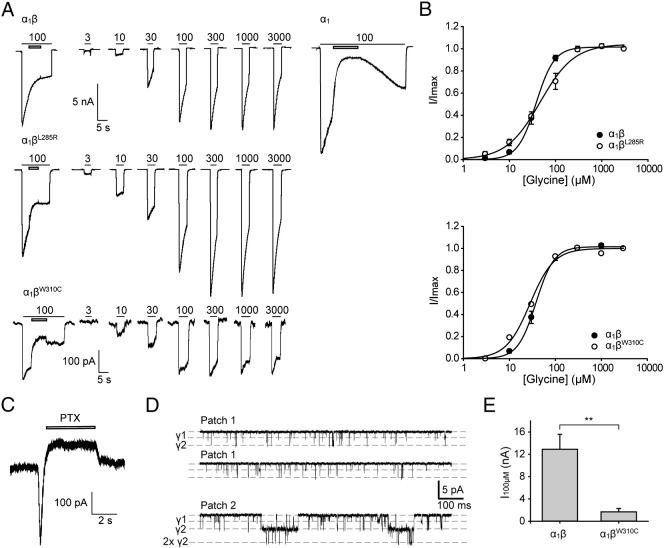
Functional characterization of human GlyRs containing β^L285R^ and β^W310C^ using electrophysiology. (A) Sample glycine dose–response traces of α_1_β, α_1_β^L285R^ and α_1_β^W310C^ GlyRs. Filled bars indicate the applied glycine concentrations in μM and non-filled bars the application of 100 μM lindane. The effect of 100 μM lindane on homomeric α_1_ subunit GlyRs is included as a control. (B) Normalized glycine dose–response curves for the GlyRs shown in panel A. (C) Inhibition of leak currents in α_1_β^L285R^ GlyRs by 100 μM picrotoxin (PTX). The inward current transient was always observed (n = 5 cells). (D) Spontaneous single-channel currents for α_1_β^L285R^ GlyRs. Most openings were of 2–10 ms duration as illustrated in Patch 1. Occasionally, with multiple channels in the patch, longer activations were also observed, as in Patch 2. (E) Mean currents activated by 100 μM glycine in cells expressing α_1_β GlyRs in a 1:1 ratio relative to those in cells expressing α_1_β^W310C^ GlyRs in a 1:10 ratio. p-Values were calculated relative to GlyR α_1_β heteromers by unpaired *t*-test: ** p < 0.01.

Electrophysiology was subsequently used to analyze the effects of the L285R and W310C mutations on GlyR function in greater detail. Dose–response relationships confirmed that the agonist sensitivity for GlyRs containing either mutated subunit did not change relative to wild-type α_1_β heteromers (Fig. 8A, B). As in the EYFP assays, the presence of the GlyR β subunit was confirmed by applying lindane (Fig. 8A). Spontaneous activity of α_1_β^L285R^-containing GlyRs could be inhibited by the pore-blocker picrotoxin (Fig. 8C). However, due to the resistance of heteromeric α_1_β GlyRs to picrotoxin (Pribilla et al., 1994), this inhibition was typically only about 100 pA. Single-channel recordings in the absence of glycine confirmed the spontaneous activity of α_1_β^L285R^ GlyRs (Fig. 8D). Averaged over four patches, mean open probability in the absence of applied glycine was 0.04 ± 0.01. The maximal unitary conductance level (denoted by γ2 in Fig. 8D) was 49.4 ± 0.7 pS with an additional sub-conductance level (γ1) at 23.9 ± 0.3 pS (both n = 4 patches). The dominant 49 pS level corresponds closely to that observed in wild-type α_1_β GlyRs (44 pS) (Bormann et al., 1993), indicating that the L285R substitution does not affect single-channel conductance. To investigate the properties of α_1_β^W310C^ GlyRs, whole-cell recordings were made from > 200 EGFP-expressing cells that had been transfected with both α_1_ and β^W310C^ subunits in a 1:10 molar ratio. Only 20 of these cells expressed glycine-gated currents, with only two of the 20 expressing lindane-insensitive GlyRs, suggesting a predominance of α_1_β^W310C^ GlyRs in the cell membrane. In both of these cells, the maximal current amplitude was significantly reduced relative to wild-type GlyRs (Fig. 8E), suggesting a reduction in either maximum open probability, single-channel conductance, and/or the number of functional channels expressed in the plasma membrane. Based on the biotinylation results, we can conclude that the reduced current amplitude is at least partly caused by reduced cell-surface expression. The properties of whole-cell currents mediated by α_1_β^L285R^ and α_1_β^W310C^ GlyRs are summarized in Table 3.

**Fig. 9:**
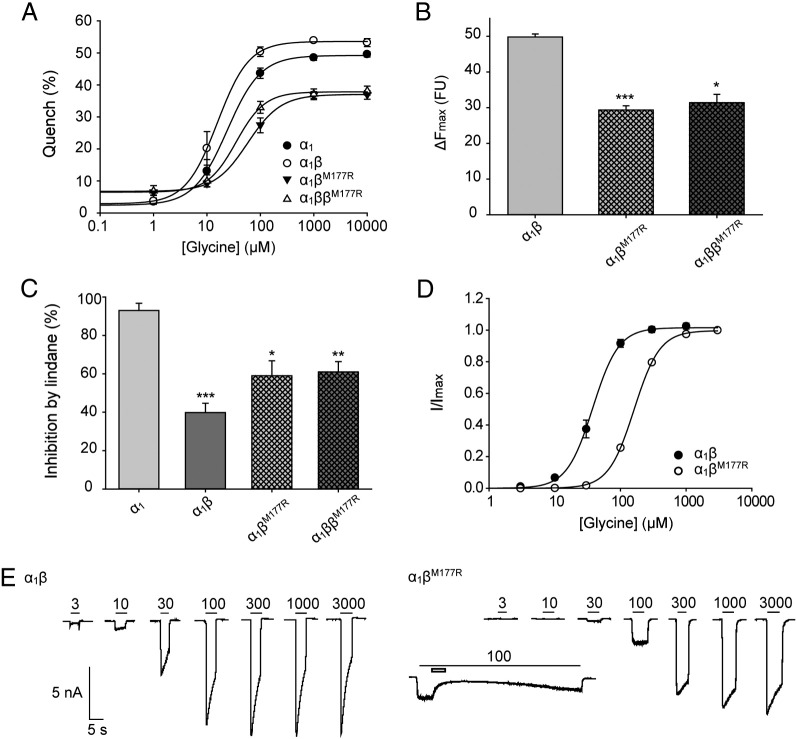
Functional characterization of human GlyRs containing β^M177R^ using the EYFP assay and electrophysiology. (A) Mean glycine dose–response curves for α_1_, α_1_β, α_1_β^M177R^ and α_1_ββ^M177R^ GlyRs using the EYFP assay. The percentage quench is plotted against the applied glycine concentration (μM). Averaged parameters of best fit to dose–response curves are summarized in Table 3. All data points represent the average quench from four experiments comprising three wells each with > 200 cells per well. (B) Maximal changes in fluorescence. ΔF_max_ is the initial fluorescence value minus the final fluorescence value and is represented in fluorescence units. p-Values were calculated relative to α_1_β GlyR heteromers by unpaired *t*-test: * p < 0.05, *** p < 0.001. (C) Inhibition by 100 μM lindane. The inhibition of currents activated by 100 μM glycine by 100 μM lindane is represented as a percentage reduction of the control maximal fluorescent quench. p-Values were calculated relative to GlyR α_1_ homomers by unpaired *t*-test: ** p < 0.01, *** p < 0.001. (D) Normalized glycine dose–response results using whole-cell patch-clamp electrophysiology for the GlyRs shown in panel E. The normalized maximal currents of cells transfected with the indicated plasmid vectors are plotted against the applied glycine concentration (μM). (E) Sample glycine dose–response traces. Filled bars indicate the applied glycine concentration in μM and non-filled bars the application of 100 μM lindane.

The M177R mutant resulted in both a reduction of maximal fluorescence quench (Fig. 9A, B) and a rightward shift in the EC_50_ value for glycine (Fig. 9A) from 17 ± 5 μM for wild-type α_1_β GlyRs to 45 ± 14 μM α_1_ββ^M177R^ and 64 ± 13 μM for α_1_β^M177R^ GlyRs (Table 4). Heteromeric α_1_β^M177R^ GlyRs exhibited a slightly reduced sensitivity to lindane (Fig. 9C). However, the reduced glycine sensitivity indicates that receptors incorporating the β^M177R^ subunit are expressed at the cell surface. Using patch-clamp electrophysiology, we confirmed that the EC_50_ value of α_1_β^M177R^ GlyRs was significantly increased from 39 ± 4 μM for wild-type α_1_β GlyRs to 162 ± 3 μM for α_1_β^M177R^ GlyRs (Fig. 9D, E) and that peak current magnitudes relative to wild-type α_1_β heteromers were significantly reduced (Fig. 9D, E, Table 3). We infer from these results that β^M177R^ was incorporated efficiently into functional heteromeric GlyRs. However, incorporation of β^M177R^ did not render GlyRs completely insensitive to lindane (Fig. 9E, inset).

## Discussion

This study reports the identification and functional characterization of novel mutations in the GlyR β subunit gene (*GLRB*) that reveal new pathogenic mechanisms underlying startle disease/hyperekplexia. We identified two new missense variants in *GLRB* causing L285R and W310C substitutions in membrane spanning domains M2 and M3. Using molecular modeling, cell-surface biotinylation assays, an EYFP-based anion flux assay and patch clamp electrophysiology, we were able to establish the likely pathogenic mechanisms for L285R and W310C, as well as a third mutation – M177R – recently reported (Al-Owain et al., 2012) in a large Saudi Arabian family with hyperekplexia. L285R is a *de novo* mutation, i.e. not found in either parent, which reduces peak current magnitude by reducing the cell-surface trafficking of GlyRs. Since the L285R substitution disrupts the integrity of the 9′ leucine hydrophobic girdle that stabilizes the channel closed state (Chang and Weiss, 1998, 1999), it was perhaps inevitable that α_1_β^L285R^ GlyRs were also associated with leak conductance indicating tonic channel opening. Since we recently described a dominant GlyR α_1_ subunit mutation in the large extracellular domain (Y128C) with a leak conductance (Chung et al., 2010), this pathogenic mechanism is an emerging theme in startle disease. Introduction of the pore-lining 9′ arginine residues might also have been expected to affect unitary conductance (Cymes and Grosman, 2011). However, we found that the dominant maximum conductance level of α_1_β^L285R^ GlyRs (49 pS) was similar to that of α_1_β GlyRs (44 pS) (Bormann et al., 1993).

On first impressions, W310C also appeared to be a classical recessive hyperekplexia mutation, interfering with the formation of cell-surface GlyRs, rather than affecting glycine sensitivity. However, detailed analysis revealed both novel pathogenic mechanisms and mode of inheritance for this missense mutation. Molecular modeling revealed that W310 is a key residue involved in a hydrophobic stack formed by aromatic residues in M1, M3 and M4 that determines GlyR subunit stoichiometry (Haeger et al., 2010). The W310C substitution in M3 is predicted to act by destabilizing intramembrane packing of α-helices, a result that is in accord with our functional studies, which show compromised cell-surface expression of α_1_β^W310C^ and α_1_ββ^W310C^ GlyRs and the formation of a significant proportion of GlyR α_1_ subunit homomers even in the presence of a ten-fold excess of β^W310C^. However, we cannot rule out the possibility that reductions in channel open probability or single-channel conductance may also have contributed to the reduced current-carrying capacity of these mutant receptors. The inheritance of this mutation is of significant clinical interest, since mild startle symptoms were reported in both parents. This mutation is likely to represent a case of incomplete dominance, i.e. a mutation that has an intermediate effect in heterozygous carriers. This mechanism is supported by our functional data, since the maximal quench was significantly reduced for both α_1_β^W310C^ and α_1_ββ^W310C^ GlyRs compared to wild-type α_1_β GlyRs. It is tempting to speculate that mutations such as W310C in *GLRB* might form the genetic basis for ‘minor startle’, an excessive startle response in the absence of hypertonia. This phenomenon has been reported (Tijssen et al., 1995, 1996, 2002) in several hyperekplexia studies, but even though ‘major’ and ‘minor’ startle can occur together in the same family, the genetic basis remains unresolved and it has been assumed by some to be a ‘normal but pronounced startle response’. Our study suggests that certain GlyR mutations may show incomplete dominance, providing a potential genetic explanation for mild startle in heterozygous carriers. This mild startle is predicted to be more common for *GLRB* mutations, since the likelihood of a heterozygous carrier being able to synthesize fully wild-type GlyRs with a 2α_1_:3β stoichiometry when carrying a defective *GLRA1* allele is 1 in 4, versus 1 in 8 for a defective *GLRB* allele.

The remaining *GLRB* substitution we studied in detail was M177R, which both molecular modeling and functional studies suggest does not disrupt cell-surface trafficking, but interferes indirectly with agonist binding, by disrupting a local β-sheet fold in the large extracellular domain. M177 is located just five residues away from F182, which participates in a cation-π interaction with glycine (Pless et al., 2008). Thus, the pathogenic mechanism for M177R resembles that of a previously reported mutation G229D, which also displayed a reduced EC_50_ for glycine (Rees et al., 2002).

Our ongoing genetic screening program has recently revealed numerous recessive mutations in the genes encoding the GlyR α_1_ subunit (*GLRA1*) and GlyT2 gene (*SLC6A5*). However, one conundrum is why so few *GLRB* mutations have been reported in startle disease. In part, this may be historical: *GLRA1* was the first startle disease gene to be discovered in 1993 (Shiang et al., 1993) and mutations in this gene explain the majority of cases of dominant hyperekplexia. By contrast, mutations in *GLRB* and *SLC6A5* were first reported in 2002 and 2006, respectively (Rees et al., 2002, 2006). This ‘head start’ for *GLRA1* is reflected in the NCBI gene-testing registry. Of the clinical laboratories world-wide offering screening for startle disease, seven screen for *GLRA1* mutations, three offer screening for *GLRB* and only one offers screening for *SLC6A5* mutations. However, this testing does not reflect the true prevalence of disease alleles — for example, we recently reported twenty new pathogenic sequence variants for GlyT2 in 17 index cases (Carta et al., 2012), as well as a new dominant GlyT2 mutation found in multiple families (Giménez et al., 2012). These studies certainly suggest that *SLC6A5* is a major startle disease gene. Methodology may also play a role: initial attempts at screening *GLRB* used single-strand conformation polymorphism (SSCP) or bi-directional di-deoxy fingerprinting (ddF) methods (Milani et al., 1998; Rees et al., 2002), which have variable sensitivity for mutation detection. By contrast, our study and two other recent reports (Al-Owain et al., 2012; Lee et al., 2012) have used Sanger DNA sequencing – currently the method of choice for mutation detection – although this is likely to be superseded in time by targeted next-generation sequencing panels (Lemke et al., 2012). The extended clinical phenotype in these cases, encompassing breathing difficulties/severe neonatal apnea episodes, bradycardia and developmental delay is likely to be explained by the loss of multiple synaptic GlyR subtypes *in vivo*, i.e. α_2_β, α_3_β as well as the predominant α_1_β isoform. In this regard, it is noteworthy that GlyR α_3_ subunit knockout mice were recently shown to have an irregular respiratory rhythm, due to loss of 5HT_1A_-mediated modulation of GlyRs in the brainstem (Manzke et al., 2010).

It is also clear that there are key differences in the relative damage caused by equivalent missense changes in GlyR α_1_ versus GlyR β subunits. For example, dominant startle disease mutations in *GLRA1* predominantly affect key amino acid residues in the M2 domain and M2-M3 linker region of the GlyR α_1_ subunit (Fig. 1*A*), where they act to uncouple ligand-binding from channel gating. Shan et al. (2011) recently demonstrated that GlyR function is less sensitive to hyperekplexia-mimicking mutations introduced into the M2–M3 loop of the GlyR β subunit than the α_1_ subunit. This suggests that the GlyR α_1_ M2–M3 loop dominates the β subunit in gating heteromeric α_1_β GlyRs and in turn that it is perhaps unlikely that a set of equivalent dominant mutations in the M2–M3 linker (Fig. 1*A*) will be found in *GLRB*. This is consistent with our finding that GlyRs containing the L285R mutant exhibit an EC_50_ that is similar to wild-type, confirming that the β subunit is not the major mediator of signal transduction in heteromeric GlyRs. Rather, the *GLRB* mutations found to date appear to cluster near key glycine binding residues (M177R, G229D) or are found in membrane-spanning domains M1–M3 (Fig. 1B), where they disrupt receptor trafficking and affect ion-channel function (L285R) or hydrophobic side-chain stacking (W310C).

Other potential pathogenic mechanisms for *GLRB* include protein truncation (via deletion, frameshift, splice site or nonsense mutations) or mutations in the gephyrin-binding site located between M3 and M4 (Fig. 1*B*). At least *in vitro*, artificial missense mutations affecting single amino acids, such as F398A in the GlyR β subunit gephyrin-binding motif are capable of significantly impairing GlyR β-gephyrin interactions (Kim et al., 2006), which would in turn abolish GlyR clustering at synapses. Based on our previous experience with *GLRA1* and *SLC6A5*, we consider it likely that a number of recessive SNVs remain to be discovered in *GLRB*. Based on the discovery of four new *GLRB* variants associated with hyperekplexia in this year alone (Al-Owain et al., 2012; Lee et al., 2012, this study), we recommend that *GLRB* should have equal status alongside *GLRA1* and *SLC6A5* in the molecular genetic diagnosis of startle disease.

The following are the supplementary data related to this article.

Supplementary table 1 PCR primers for GLRB exon amplification.

## Competing interests

All authors have declared that no competing interests exist.

## Figures and Tables

**Table 1:** Single nucleotide variants in *GLRB* associated with startle disease/hyperekplexia. Mutations G229D/IVS5+5G>A, M177R, and S321F were reported previously (Al-Owain et al., 2012; Lee et al., 2012; Rees et al., 2002). Panel: DNA sequence electropherograms for the two novel mutations (c.920_921ΔInsGA/L285R and c.G996T/p.W310C) reported in this study. Bioinformatics analysis was conducted using SIFT (Kumar et al., 2009) with ENSP00000264428 and Polyphen-2 (Adzhubei et al., 2010).

Position	Exon	Protein precursor/mature	Type	SIFT	Polyphen-2	Overall prediction
c.T596G	6	M199R/M177R	Missense	Damaging (0)	Possibly damaging (0.802)	Pathogenic
c.G752A	8	G251D/G229D	Missense	Damaging (0)	Probably damaging (1.0)	Pathogenic
IVS5+5G>A	–	–	Splice site	–	–	Pathogenic
c.920_921ΔInsGA	9	L307R/L285R	Missense	Damaging (0)	Probably damaging (0.997)	Pathogenic
c.G996T	9	W332C/W310C	Missense	Damaging (0)	Probably damaging (0.999)	Pathogenic
c.C1028T	9	S343F/S321F	Missense	Damaging (0)	Probably damaging (0.990)	Pathogenic


**Table 2:** Properties of human GlyRs containing β^L285R^ and β^W310C^ subunits using the EYFP assay. EC_50_ values, Hill coefficients (n_H_), the maximal fluorescence changes (ΔF_max_), the maximal percentages of quenched cells (cells_quench_) and the inhibition of 10 μM glycine currents by 100 μM lindane (LIN_inh_) are represented. p-Values were calculated relative to GlyR α_1_β subunit heteromers except for inhibition by lindane, which was compared to GlyR α_1_ subunit homomers by unpaired *t*-test.

	EYFP assay					
EC_50_ (μM)	n_H_	ΔF_max_ (FU)	cells_quench_ (%)	LIN_inh_ (%)	n
α_1_	8 ± 1	1.7 ± 0.1	44 ± 1	94 ± 2	111 ± 4	12
α_1_β	5 ± 1	3.9 ± 2.2	44 ± 2	96 ± 2	38 ± 2⁎⁎⁎	12
α_1_β^L285R^	4 ± 1	0.9 ± 0.1⁎	19 ± 2⁎⁎⁎	86 ± 7	6 ± 7⁎⁎⁎	12
α_1_ββ^L285R^	4 ± 1	0.9 ± 0.1⁎	19 ± 2⁎⁎⁎	83 ± 5	14 ± 7⁎⁎⁎	12
α_1_β^W310C^	13 ± 2⁎	4.3 ± 1.8	32 ± 3⁎	81 ± 5⁎	51 ± 10⁎⁎	12
α_1_ββ^W310C^	11 ± 1⁎⁎	5.1 ± 1.5	32 ± 1⁎⁎	90 ± 1⁎	32 ± 12⁎⁎⁎	12

⁎ p < 0.05.

⁎⁎ p < 0.01.

⁎⁎⁎ p < 0.001.

**Table 3:** Properties of α_1_β, α_1_β^L285R^, α_1_β^W310C^ and β^M177R^ GlyRs using whole-cell patch-clamp electrophysiology. EC_50_ values, Hill coefficients (n_H_) and the maximal currents (I_max_) are represented. p-Values were calculated relative to the wild-type α_1_β subunit GlyRs by unpaired *t*-test.

	Whole-cell patch-clamp electrophysiology			
EC_50_ (μM)	n_H_	I_max_ (nA)	n
α_1_β	39 ± 4	2.4 ± 0.2	14 ± 3	5
α_1_β^L285R^	49 ± 7	1.2 ± 0.1⁎⁎	9 ± 4	3
α_1_β^W310C^	28, 58	1.7, 4.0	0.22, 0.15	2
α_1_β^M177R^	162 ± 3⁎⁎⁎	2.2 ± 0.1	8 ± 1	5

⁎⁎ p < 0.01.

⁎⁎⁎ p < 0.001.

**Table 4:** Properties of α_1_β^M177R^ GlyRs using the EYFP assay. EC_50_ values, Hill coefficients (n_H_), the maximal fluorescence changes (ΔF_max_), the maximal percentages of quenched cells (cells_quench_) and the inhibition of 100 μM glycine currents by blocking with 100 μM lindane (LIN_inh_) are represented. p-Values were calculated relative to GlyR α_1_β subunit heteromers except for inhibition by lindane, which was compared to GlyR α_1_ subunit homomers by unpaired *t*-test.

	EYFP assay					
EC_50_ (μM)	n_H_	ΔF_max_ (FU)	cells_quench_ (%)	LIN_inh_ (%)	n
α_1_	25 ± 5	1.5 ± 0.1	46 ± 2	97 ± 1	92 ± 5	12
α_1_β	17 ± 5	1.7 ± 0.1	50 ± 1	96 ± 1	42 ± 6⁎⁎⁎	12
α_1_β^M177R^	64 ± 13⁎	3.4 ± 1.9	29 ± 1⁎⁎⁎	81 ± 2⁎⁎⁎	54 ± 8⁎	12
α_1_ββ^M177R^	45 ± 14	3.4 ± 1.9	31 ± 2⁎	84 ± 2⁎⁎⁎	60 ± 7⁎⁎	12

⁎ p < 0.05.

⁎⁎ p < 0.01.

⁎⁎⁎ p < 0.001.
