# Supplementary material for: Novel missense mutations in the glycine receptor β subunit gene (GLRB) in startle disease
Source: Neurobiol Dis. 2013 Apr;52:137–49. doi: 10.1016/j.nbd.2012.12.001 (PMC3581774; doi:10.1016/j.nbd.2012.12.001)
Supplement: Supplementary table 1 — PCR primers for GLRB exon amplification. [file mmc1.doc]

Table 1: PCR primers for *GLRB* exon amplification

| Exon 1 | forward | **TGTAAAACGACGGCCAGT**CTGTCGGGACACCTCTTC |
| --- | --- | --- |
|  | reverse | **CAGGAAACAGCTATGACC**TTCCGCAAAGGCGTCTATTC |
| Exon 2 | forward | **TGTAAAACGACGGCCAGT**TTTGGGTAGTAATGCTTATACTC |
|  | reverse | **CAGGAAACAGCTATGACC**CTTCATAAACCACCTTACTT |
| Exon 3 | forward | **TGTAAAACGACGGCCAGT**GCAGTCTTTCCTCCCAATGA |
|  | reverse | **CAGGAAACAGCTATGACC**AAACCCATTGCGGATGT |
| Exon 4 | forward | **TGTAAAACGACGGCCAGT**ACATGTTATACTGAGACCATAGAT |
|  | reverse | **CAGGAAACAGCTATGACC**TATACTAGGAAAGGGCATGGATTA |
| Exon 5-6 | forward | **TGTAAAACGACGGCCAGT**GGGCCGAATAAGTTCTTAAA |
|  | reverse | **CAGGAAACAGCTATGACC**AGTAGAATGTATTACCCAGAC |
| Exon 7 | forward | **TGTAAAACGACGGCCAGT**TGACCATGGGTTTTACCTCT |
|  | reverse | **CAGGAAACAGCTATGACC**CTACTCTCCCCTTGGCT |
| Exon 8 | forward | **TGTAAAACGACGGCCAGT**ACCGTTTCCAGGTCTGTC |
|  | reverse | **CAGGAAACAGCTATGACC**ACACCGAAATTGGTGGCTA |
| Exon 9 | forward | **TGTAAAACGACGGCCAGT**TTGTTCATGGGTCATTGGAAGTTA |
|  | reverse | **CAGGAAACAGCTATGACC**CACATACACAAAGGTGTCT |
| Exon 10 | forward | **TGTAAAACGACGGCCAGT**CGTTTGAAGAGATGTGT |
|  | reverse | **CAGGAAACAGCTATGACC**TCCAAAATGCAATGTTGCTA |
